# Implications of mutational spectrum in myelodysplastic syndromes based on targeted next-generation sequencing

**DOI:** 10.18632/oncotarget.19628

**Published:** 2017-07-27

**Authors:** Yuanyuan Xu, Yan Li, Qingyu Xu, Yuelong Chen, Na Lv, Yu Jing, Liping Dou, Jian Bo, Guangyuan Hou, Jing Guo, Xiuli Wang, Lili Wang, Yonghui Li, Chongjian Chen, Li Yu

**Affiliations:** ^1^ Department of Hematology and BMT center, Chinese PLA General Hospital, Beijing 100853, China; ^2^ Department of Hematology, Hainan Branch of Chinese PLA General Hospital, Sanya 572013, Hainan Province, China; ^3^ Medical school of Nankai University, Tianjin 300071, China; ^4^ Annoroad Gene Technology Co. Ltd, Beijing 100176, China; ^5^ Department of Hematology, General Hospital of Shenzhen University, Shenzhen 518060, China

**Keywords:** myelodysplastic syndromes, mutational spectrum, next-generation sequencing, risk stratification

## Abstract

Myelodysplastic syndromes (MDS) are a group of myeloid hematological malignancies, with a high risk of progression to acute myeloid leukemia (AML). To explore the role of acquired mutations in MDS, 111 MDS-associated genes were screened using next-generation sequencing (NGS), in 125 patients. One or more mutations were detected in 84% of the patients. Some gene mutations are specific for MDS and were associated with disease subtypes, and the patterns of mutational pathways could be associated with progressive MDS. The patterns, frequencies and functional pathways of gene mutations are different, but somehow related, between MDS and AML. Multivariate analysis suggested that patients with ≥ 2 mutations had poor progression-free survival, while *GATA1/GATA2*, *DNMT3A* and *KRAS/NRAS* mutations were associated with poor overall survival. Based on a novel system combining IPSS-R and molecular markers, these MDS patients were further divided into 3 more accurate prognostic subgroups. A panel of 11 target genes was proposed for genetic profiling of MDS. The study offers new insights into the molecular signatures of MDS and the genetic consistency between MDS and AML. Furthermore, results indicate that MDS could be classified by mutation combinations to guide the administration of individualized therapeutic interventions.

## INTRODUCTION

Myelodysplastic syndromes (MDS) are a group of myeloid hematological malignancies, characterized by varying degrees of cytopenias and a high risk of progression to acute myeloid leukemia (AML) [1, 2]. Due to the conspicuous clinical and biological heterogeneity, an optimized treatment, based on accurate diagnosis and prognostic evaluation for individual patients, is particularly important [3]. The International Prognostic Scoring System (IPSS) and other models have been used for these purposes [3, 4], and the subsequent revision of the IPSS (IPSS-R) further improved the evaluation [5].

Recently, next-generation sequencing (NGS) has successfully been used to determine mutational profiles of different types of cancer, due to its massive parallel sequencing ability and high throughput multiplexing capacity [6]. Characterizing recurrent functional somatic mutations by targeted NGS aids in identifying disease-associated mutations, which is particularly relevant for clinical practice [7]. Currently, knowledge of the molecular pathogenesis of MDS has dramatically improved due to the identification of major mutational targets [8]. These mutations affect genes involved in DNA methylation (*DNMT3A, TET2, IDH1/IDH2*), chromatin modification (*EZH2, ASXL1*), transcription (*RUNX1, GATA1/GATA2*), RNA splicing (*SF3B1, U2AF1, SRSF2* and *ZRSR2*) and signal transduction (*JAK2, KRAS/NRAS, CBL*) [9–17]. However, the list of gene mutations implicated in the pathogenesis of MDS is still growing [18–20]. Two large studies from the United Kingdom and Japan have discovered oncogenic mutations in 78% and 89.5% of patients with MDS using targeted sequencing and have developed novel prognostic models using molecular sequencing data [21, 22]. Other studies have suggested that the mutational status of multiple gene targets could better predict the clinical outcome of MDS [23, 24], implying that targeted sequencing could offer a cost-effective, front-line diagnostic tool for MDS. In line with this, the European Leukemia Network and a Clinical Advisory Committee have suggested that a conclusive diagnosis and a reliable prognostic evaluation should be performed based on the new development and discovery of gene mutations in MDS, which is being considered by the World Health Organization (WHO) for a revised classification of risk-groups for MDS [20, 25].

Therefore, we investigated the mutational signature of MDS in a Chinese patient-population using targeted NGS to detect whether there are different spectrums of genetic mutations. In addition, this study was aimed at indentifying the genetic differences and relationship between MDS and AML, and integrating the existing results on targeted mutations in MDS into a new gene panel with promising clinical applications.

## RESULTS

### Landscape of gene mutations

**Figure 1:**
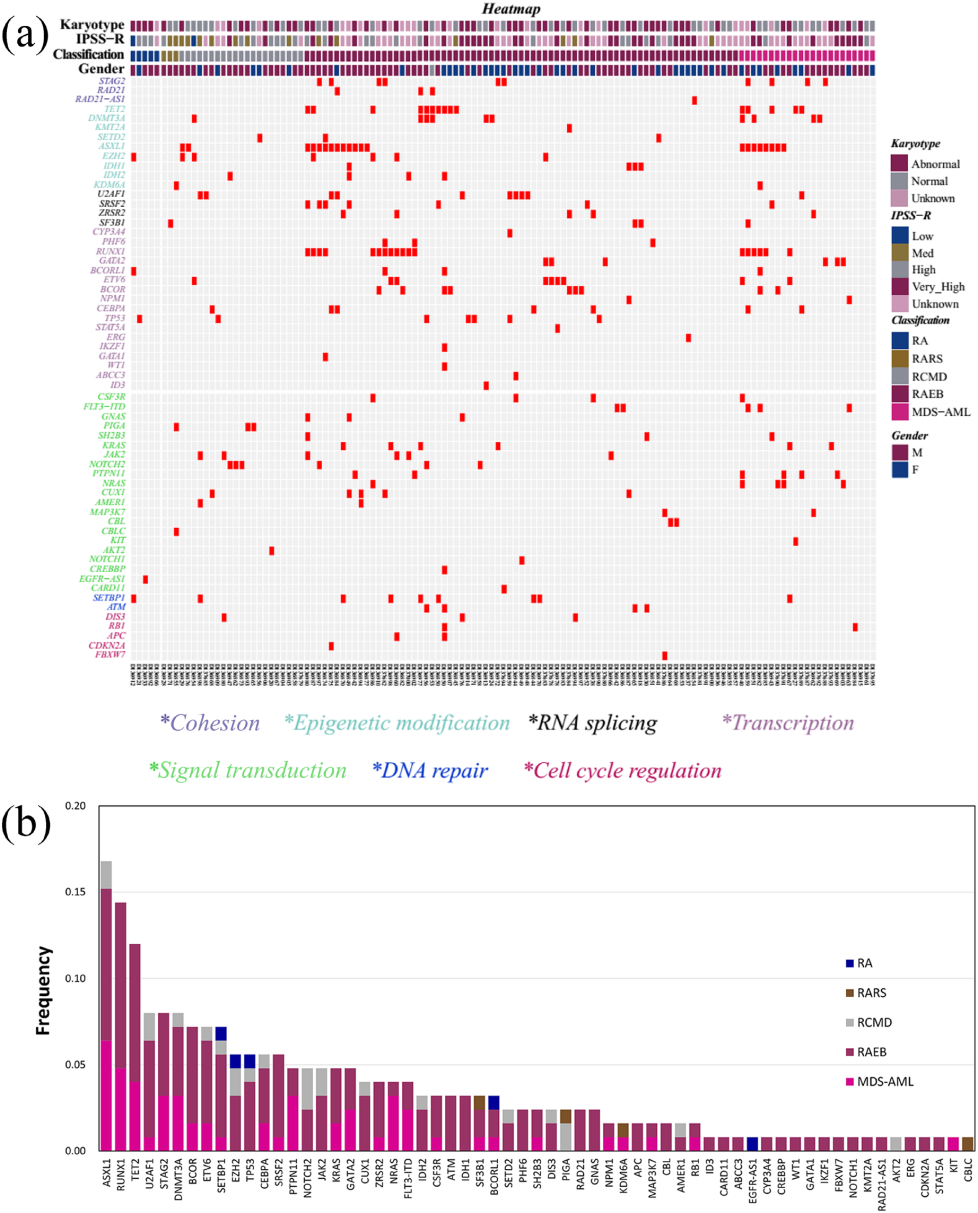
The genomic architecture of MDS **(a)** Distribution of mutations in 125 MDS patients. Red boxes indicate mutations. **(b)** Frequencies of mutations identified in the cohort of 125 subjects, divided according to MDS subtype.

The sequencing depth ranged between 200× and 1897×, and the median depth was 861× (Supplementary Figure 1). The detailed NGS data generation of the 125 patients was in the Supplementary Table 1. In total, 1491 single-nucleotide variants (SNVs) and 701 small insertions and deletions were detected in the target regions, in 125 patients. We focused on the mutations with altered amino acids in coding regions. Steps were taken to remove germline and harmless mutations (Supplementary Methods). A landscape of gene aberrations was generated with respect to clinical information and mutations classified by functional categories (Figure 1a). Consequently, 308 mutations in 61 genes were discovered in 105 of 125 cases (84.0%) (Figure 1b, Supplementary Table 2). The average number of mutations per case was 2.46 (308/125), whereas the number in our previous AML study was 3.60 (342/95) [26]. This may indicate that more mutations are acquired during the translation from MDS to AML.

### Patterns of genomic lesions

The mutation positions and types of a subset of the 61 genes were shown in Supplementary Figure 2. The base change data in the forward direction was collected for analyses. Nucleotide substitution was the main mutation type (67.9%, 209/308), and the proportions of transitions and transversions were 60.1% (126/209) and 39.7% (83/209), respectively, indicating a predominance of transitions. The respective proportions were 74.6% (167/224), 65.9% (110/167) and 34.1% (57/167) in our previous AML sequencing data [26], indicating a consistency in genomic lesions between MDS and AML (p = 0.094, p = 0.266, p = 0.266, respectively). Interestingly, the C→T transition was the most prevalent nucleotide substitution in MDS, with a significantly higher frequency than in AML (26.3% vs. 15.0%, p = 0.008), whereas the G→A transition was the most prevalent nucleotide substitution in AML with a significantly higher frequency than in MDS (33.7% vs. 22.5%, p = 0.001) [26]. These results suggest that the genomic lesion patterns identified in MDS and AML were both related and different.

### Frequency and spectrum of gene mutations

The frequency of each detected gene abnormality is shown in Figure 1b. Of the 61 identified genes, 13 were mutated in more than 5% of MDS subjects. The frequency of mutations was highest in *ASXL1* (16.8%), followed by *RUNX1* (14.4%) and *TET2* (12.0%). The overall distribution of mutations observed in the cohort was mirrored within the categories of MDS (Figure 1b). Mutations in genes other than *PIGA*, *EGFR-AS1*, *AKT2* and *CBLC*, were largely distributed in subjects with refractory anemia with excess blasts (RAEB) (n = 56) or the AML with multilineage dysplasia following MDS (MDS-AML) (n = 24). Mutation frequencies of 2 (*ASXL1* and *TET2*) of the top 3 genes with > 10% frequency in MDS (*ASXL1, RUNX1* and *TET2*) were similar to those in AML [26]. For *RUNX1,* the mutation frequency was higher in MDS than in AML (18/125, 14.4% *vs.* 5/95, 5.3%). More genes with > 10% mutation frequency (including *CEBPA*, *NPM1*, *DNMT3A*, *FLT3-ITD*, *NRAS*, *IDH2* and *WT1*) were found in AML compared to MDS. Specifically, the mutation frequencies of *NPM1*, *FLT3-ITD*, *NRAS*, *IDH2* and *WT1* were < 5% in MDS. The results suggest that there is consistency and heterogeneity in the spectrum of high-frequency mutated genes between MDS and AML.

### Specificity of mutations in MDS

**Figure 2:**
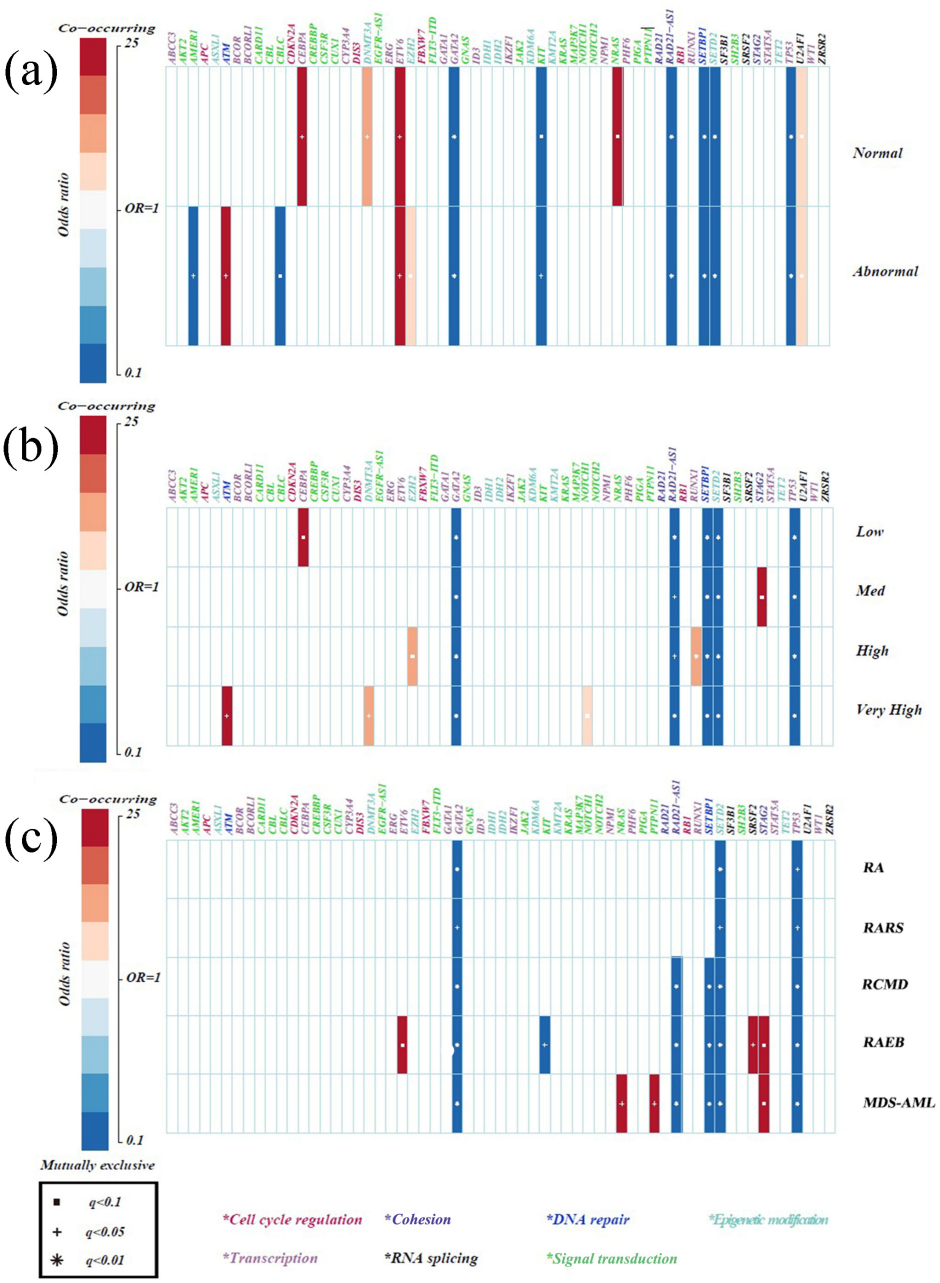
Specificity of mutations in MDS compared with healthy donators **(a)** Correlations between gene mutations and chromosomes. **(b)** Correlations between gene mutations and IPSS-R risk stratifications. **(c)** Correlations between gene mutations and WHO classifications. Only those associations with a q value (false discovery rate adjusted *p* value) < 0.1 were shown. Associations are colored by odds ratio. Red colors label genes that were co-mutated in MDS more than expected, and blue colors label mutually exclusive gene mutations in MDS. Gene names are color coded by the different functional pathways below the figure. Due to a high SNP in healthy donators, the results of mutually exclusive genes had little meaning and the co-mutated genes were emphasized.

Compared with healthy donators, some gene aberrations were associated with MDS (Figure 2). Mutations in *CEBPA* and *NRAS* were typically found in patients with normal karyotypes, followed by mutations in *DNMT3A*. Abnormal cytogenetics were closely correlated with *ATM* mutations and only very slightly related to *EZH2* mutations (Figure 2a). Notably, mutations in the transcription factor *ETV6* were associated with both normal and abnormal karyotypes. Using the IPSS-R evaluation, a strong correlation was found between *CEBPA* mutations and low risk. Patients at high risk were weakly linked to *EZH2* and *RUNX1* mutations. However, patients at the very high risk presented with *ATM* mutations, followed by *DNMT3A* and *NOTCH1* mutations (Figure 2b).

By analyzing MDS subtypes, we found the coexistence of signal pathway gene mutations (*PTPN11* and *NRAS*) in MDS-AML patients, implying different functions of the same biological pathway genes in specific genomic contexts [21]. Furthermore, *ETV6* and *SRSF2* appeared to be more frequently mutated in RAEB, whereas *STAG2* mutations frequently occurred in both RAEB and MDS-AML, suggesting consistency between the two subtypes (Figure 2c). Further analyses of the subgroups of IPSS-R and MDS subtypes demonstrated that *STAG2* and *RUNX1* mutations were indeed more frequent in cases at medium and high risk, respectively, while *NRAS* mutations were moderately associated with MDS-AML, meaning they could potentially have early diagnostic value for MDS-AML. Finally, *TP53* and *NOTCH1/NOTCH2* mutations more frequently appeared in complex karyotypes (p = 0.005, p = 0.001, respectively).

### Subtypes and disease outcome correlate with the number of gene mutations

**Figure 3:**
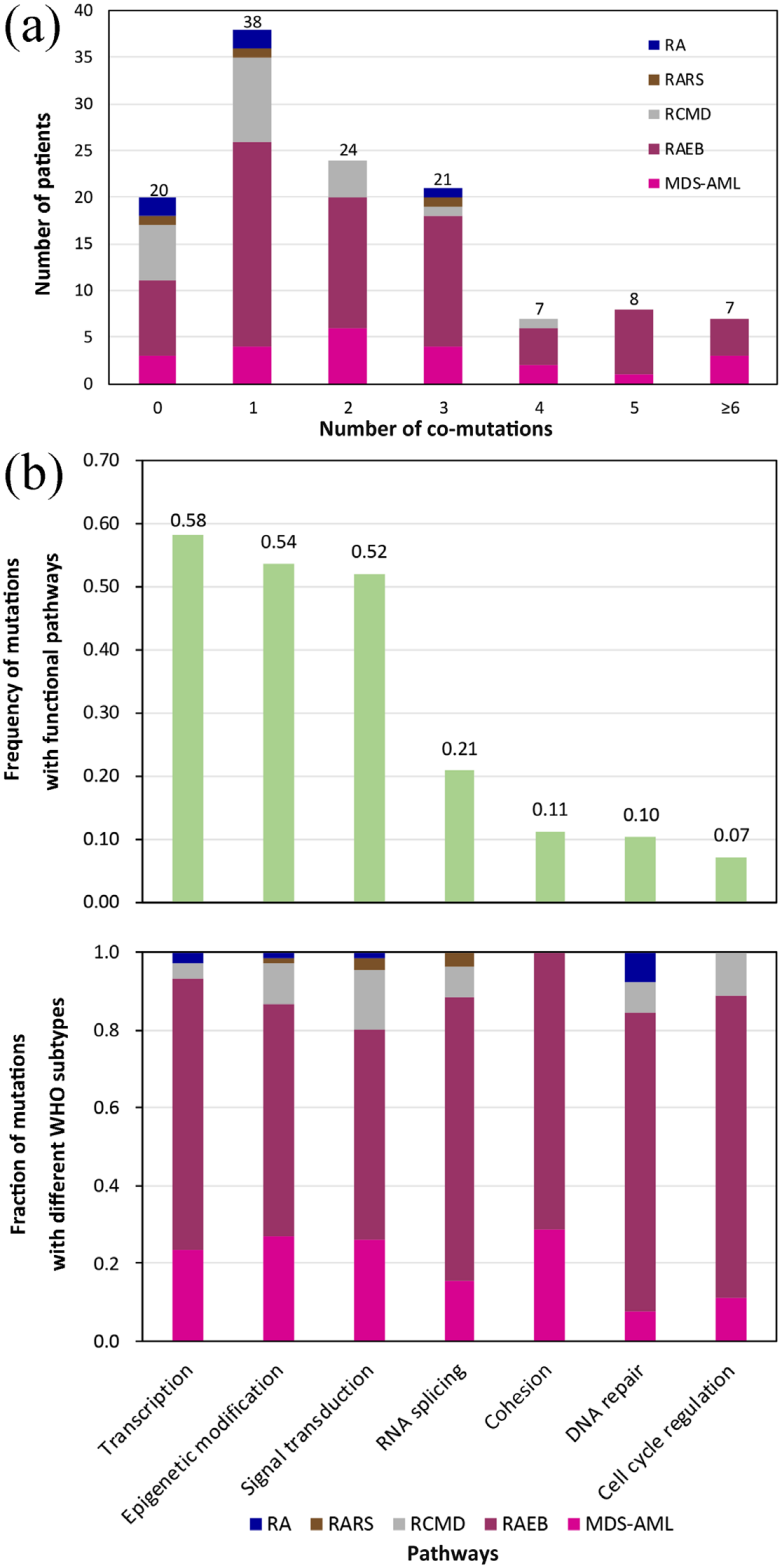
**(a)** Distribution of the number of co-occurring mutations (including point mutations and indels) relative to MDS subtypes. **(b)** The number of mutations involved in common functional pathways classified according to different WHO subtypes.

The average number of mutations *per* subtype was 1.0 (refractory anemia, RA, 5/5), 1.3 (refractory anemia with ring sideroblasts, RARS, 4/3), 1.14 (refractory anemia with multilineage dysplasia, RCMD, 24/21), 2.36 (RAEB, 172/73) and 2.70 (MDS-AML, 62/23) (p = 0.02). The median PFS was 96.00 (RCMD), 23.49 (RAEB) and 7.80 (MDS-AML) months (p < 0.01), with the 3-year PFS rates being 54.0 ± 11.3% (RCMD), 42.5 ± 7.5% (RAEB) and 1.5 ± 9.5% (MDS-AML) (p < 0.01), respectively. For this study, RA and RARS were not considered due to the limited number of cases. As shown in Figure 3a, a high proportion of patients from every MDS subtype, harbored only one gene mutation (30.4%, 38/125). Complex genetic abnormalities with ≥ 2 mutations were identified in 53.6% (67/125) of cases, most of which could be grouped in the subsets with progressive MDS (RAEB, 64.2%; MDS-AML, 23.9%).

### Gene mutations involved in common functional pathways

Considering the targeted sequencing and prognostic discrepancies among various subtypes of MDS, we further analyzed the mutation frequencies in 7 common functional pathways hypothesized to be characteristic for MDS pathogenesis (Figure 3b, Supplementary Table 3). We found that the most frequently mutated pathway was transcription, with mutations identified in 58.4% of patients, followed by epigenetic modification (53.6%), signal transduction (52.0%), RNA splicing (20.8%), cohesion (11.2%), DNA repair (10.4%) and cell cycle regulation (7.2%).

Notably, only mutations affecting epigenetic modification and signal transduction occurred in each MDS subtype. Mutations affecting cohesion only appeared in RAEB and MDS-AML, but not in RCMD. The most common mutations in RCMD were those involved in signal transduction (41.7%), but decreased significantly in RAEB (20.3%) (p = 0.02). Moreover, mutations in transcription increased from 12.5% in RCMD to a maximum of 29.7% in RAEB (p = 0.078). When comparing RAEB with MDS-AML, there was no significant difference in the ratios of mutations in each functional pathway (p > 0.1). Additionally, RAEB had a higher ratio in every subtype of identified mutations compared to MDS-AML (Figure 3b).

To explore the genetic correlation between progressive MDS (including RAEB and MDS-AML) and AML, the frequencies of different mutations between the two hematologic malignancies were compared using results from this and our previous study [26]. We found that the most common mutations in AML occurred in the transcription pathway and that these were significantly more abundant than in progressive MDS (p = 0.02). On the contrary, mutations in the DNA repair and cell cycle regulation pathways were commonly found in MDS but not in the AML cohort. The percentages of mutations in genes involved in epigenetic modification, signal transduction, RNA splicing and cohesion were similar between MDS and AML (p > 0.05).

### Clinical features of patients with mutations in DNA methylation

Considering that mutations in genes related to DNA methylation (*TET2*, *DNMT3A* and *IDH1/IDH2*) were amongst the top 10 mutations identified in progressive MDS and that hypomethylation agents (HMAs) are widely used in treating MDS, we analyzed the clinical features of the patients harboring these mutations. Of the 108 cases with follow-up data, 23 carried 26 mutations in *TET2*, *DNMT3A* and/or *IDH1/IDH2*. Except for *TET2*/*DNMT3A* co-mutations in two cases and a *TET2*/*IDH2* co-mutation in one case, all other cases carried mutations in only one of these. Compared to the 85 cases without mutations on DNA methylation, the 23 patients with mutations were older and had progressive diseases, more complex karyotypes and ≥ 2 mutations (age ≥ 60 years: 65.2% *vs.* 25.9%, p = 0.000; RAEB&MDS-AML: 91.3% *vs.* 68.2%, p = 0.027; complex karyotypes: 26.3% *vs.* 8.1%, p = 0.028; ≥ 2 mutations: 87.0% *vs.* 43.5%, p = 0.000). However, there was no significant difference in OS and PFS between these two groups (p = 0.078 and p = 0.205, respectively), which might be related to the fact that more patients with mutations in DNA methylation received treatment with HMAs (65.2% *vs*. 31.8%, p = 0.004).

### Prognostic significance of the gene mutations

Follow-up data were available for 108 patients (Table 1). To assess the prognostic significance of identified mutations, we focused on 22 genes with ≥ 5% mutation frequency (*ASXL1*, *RUNX1*, *TET2*, *U2AF1*, *STAG2*, *DNMT3A*, *ETV6*, *SETBP1*, *EZH2*, *TP53*, *CEBPA*, *SRSF2*, *BCOR/BCORL1*, *KRAS/NRAS*, *GATA1/GATA2*, *NOTCH1/NOTCH2* and *IDH1/IDH2*) and considered factors including: age (< 60 yr *vs.* ≥ 60 yr), WHO classifications (progressive MDS *vs.* non-progressive MDS), treatment strategy (HSCT *vs.* non-HSCT) as well as IPSS-R and IPSS-R-M (molecular maker).

**Table 1:** Baseline characteristics of patients (N = 125)

Demographics	N = 125
Gender	
Male	83 (66%)
Female	42 (34%)
Age	
< 60	83 (66%)
≥ 60	42 (34%)
MDS classification (WHO 2008)	N = 125
RA	5 (4%)
RARS	3 (2%)
RCMD	21 (17%)
RAEB1	21 (17%)
RAEB2	51 (41%)
MDS-AML^#^	24 (19%)
Cases with follow-up	N = 108
Median follow-up (OS/PFS)	18 Months/ 13 Months
3-year Cumulative OS	62.4 ± 5.6%
3-year Cumulative PFS	51.1 ± 5.5%
Median OS	/
Median PFS	41 Months
Blood counts at diagnosis	N = 108
Hemoglobin level	77.8 ± 20.8 g/L
Neutrophil count*	0.5 (0.3, 0.8) × 10^9^/L
Platelet count*	57.0 (21.0, 93.0) × 10^9^/L
Marrow blast (%)	N = 108
≤ 2	3 (3%)
> 2 and <5	26 (24%)
≥ 5 and ≤10	24 (22%)
> 10	55 (51%)
Cytogenetics	N = 108
Normal	43 (40%)
Abnormal	50 (46%)
Failed/not done	15 (14%)
IPSS-R risk group	N = 108
Very low	0 (0%)
Low	2 (2%)
Intermediate	15 (14%)
High	24 (22%)
Very high	35 (32%)
Unknown	32 (30%)
Therapy strategy	N = 108
Supportive	27 (25%)
HMAs	42 (39%)
HSCT without HMAs pre-treatment	32 (30%)
HSCT bridged by HMAs pre-treatment	7 (6%)

^#^ Patients had ≥ 20% blasts with a history of MDS, which is classified as AML with multilineage dysplasia following MDS according to the 2008 WHO classification.

* median (P25, P75)

**Figure 4:**
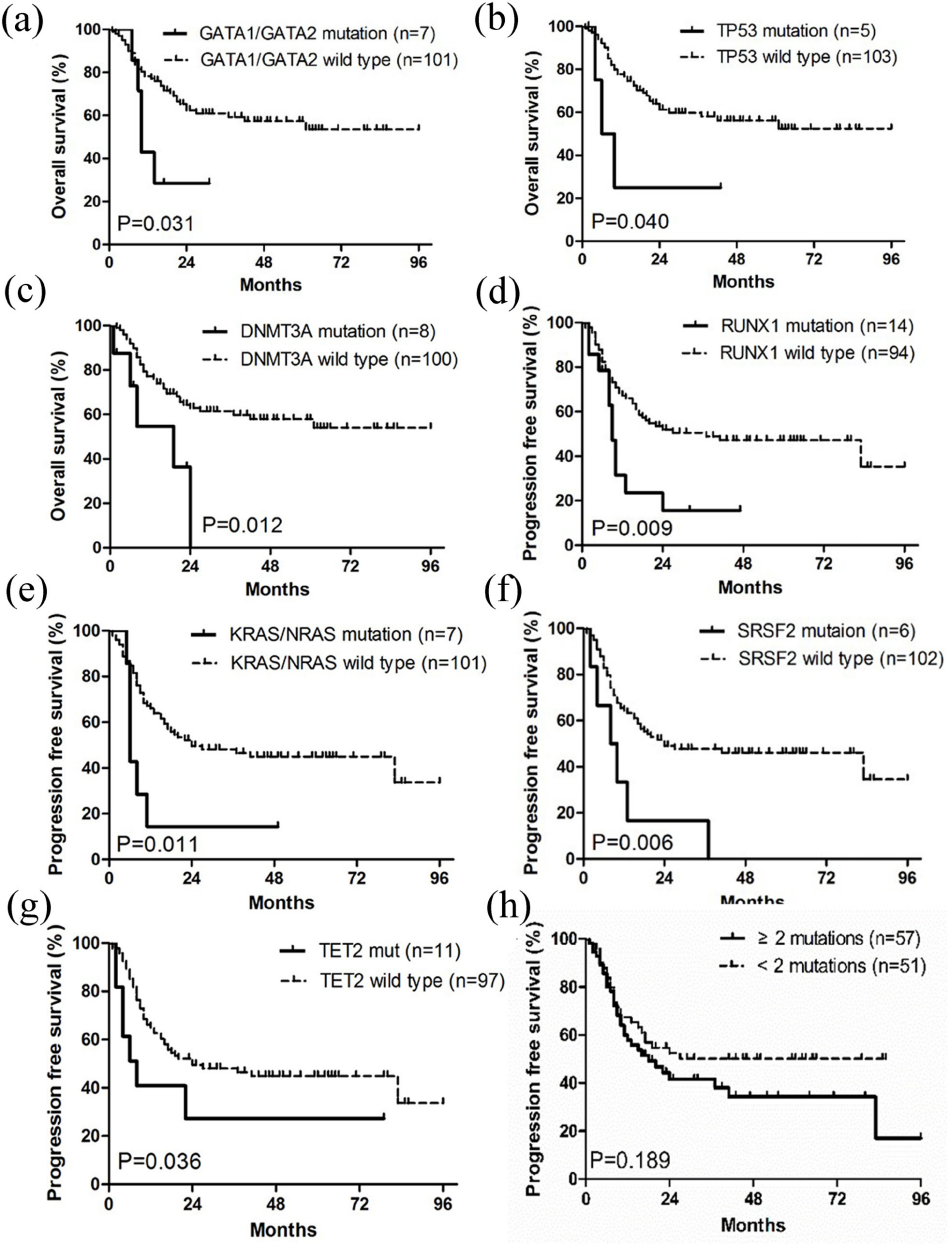
Kaplan-Meier curves of overall survival (OS) and progression free survival PFS **(a-c)** Patients with *GATA1/GATA2*, *TP53* and *DNMT3A* mutations had worse OS than wild type groups. **(d-g)** Patients with *RUNX1*, *KRAS/NRAS*, *SRSF2* and *TET2* mutations had worse PFS than wild type groups. **(h)** Patients with ≥ 2 mutations tended to have shorter PFS than those with < 2 mutations. OS and PFS were stratified by univariate prognostic factors. P values were calculated using the log-rank test.

Based on univariate analyses, the age of ≥ 60 years was unfavorable for both OS and PFS. Patients without HSCT treatment had worse OS and those with progressive MDS had shorter PFS (p < 0.05) (Table 1). Mutations (Figure 4) in *GATA1/GATA2*, *DNMT3A* and *TP53* negatively affected OS (p < 0.05), while mutations in *RUNX1*, *KRAS/NRAS*, *SRSF2* and *TET2* were associated with shorter PFS (p < 0.05). PFS also tended to be shorter in patients with complex genetic abnormalities harboring ≥ 2 mutations (p = 0.189).

Multivariate COX regression analyses, displayed in Table 2, indicated that the age of ≥ 60 years was an independent factor for both poor OS and PFS (p < 0.05). Progressive MDS, based on WHO-classification, unfavorably affected PFS independently (p < 0.05). Mutations in *KRAS/NRAS*, *GATA1/GATA2* and *DNMT3A* independently contributed to poor OS, whereas *IDH1/IDH2* mutations were associated with a relatively higher PFS. Lastly, complex genetic abnormalities with ≥ 2 mutations were an ominous sign for PFS (p < 0.05).

**Table 2:** Univariate and multivariate analyses for OS and PFS

N=108	Univariate		Multivariate^*^	
	OS (HR, 95%CI)	PFS (HR, 95%CI)	OS (HR, 95%CI)	PFS (HR, 95%CI)
Age (≥ 60 *vs.* < 60 year)	2.456 (1.340-4.504)	1.846 (1.094-3.115)	2.230 (1.192-4.170)	2.278 (1.320-3.931)
HSCT *vs.* non-HSCT	1.993 (1.021-3.888)	- -	- -	- -
Progressive MDS *vs.* non-progressive MDS)	- -	2.190 (1.127-4.258)	- -	1.819 (1.418-2.332)
GATA1/GATA2 mutation	2.703 (1.048-6.970)	- -	3.714 (1.341-10.287)	- -
*TP53* mutation	3.160 (1.117-59.320)	- -	- -	- -
*DNMT3A* mutation	3.106 (1.211-7.971)	- -	2.842 (1.070-7.547)	- -
*RUNX1* mutation	- -	2.208 (1.136-4.293)	- -	- -
KRAS/NRAS mutation	- -	2.678 (1.137-6.307)	3.525 (1.288-9.650)	- -
*SRSF2* mutation	- -	2.870 (1.225-6.722)	- -	- -
*TET2* mutation	- -	2.248 (1.103-10.060)	- -	- -
IDH1/IDH2 mutation	- -	- -	- -	0.273 (0.081-0.919)
≥ 2 mutations *vs.* <2 mutations	- -	- -	- -	3.364 (1.428-7.925)

^*^Multiple variables were selected for the Cox proportional hazard model: age (≥ 60 vs. < 60 year), white blood cell count, platelet count, hemoglobin, bone marrow blast, IPSS-R, administered treatment therapy (HSCT *vs.* non-HSCT), diagnosis (progressive MDS *vs.* non-progressive MDS) and mutations (including mutations with frequency ≥ 5% and those involved in epigenetic modification: *RUNX1*, *BCOR/BCORL1*, *U2AF1*, *KRAS/NRAS*, *GATA1/GATA2*, *ETV6*, *NOTCH1/NOTCH2*, *STAG2*, *SETBP1*, *SRSF2*, *TP53*, *CEBPA*, *ASXL1*, *TET2*, *DNMT3A*, *IDH1/IDH2*, *EZH2*, *SETD2*, *KDM6A*, *KMT2A*. And when analyzing “ ≥ 2 mutations *vs.* <2 mutations”, the mutation variables were removed.

*Abbreviations:* OS, overall survival; PFS, progression-free survival.

**Figure 5:**
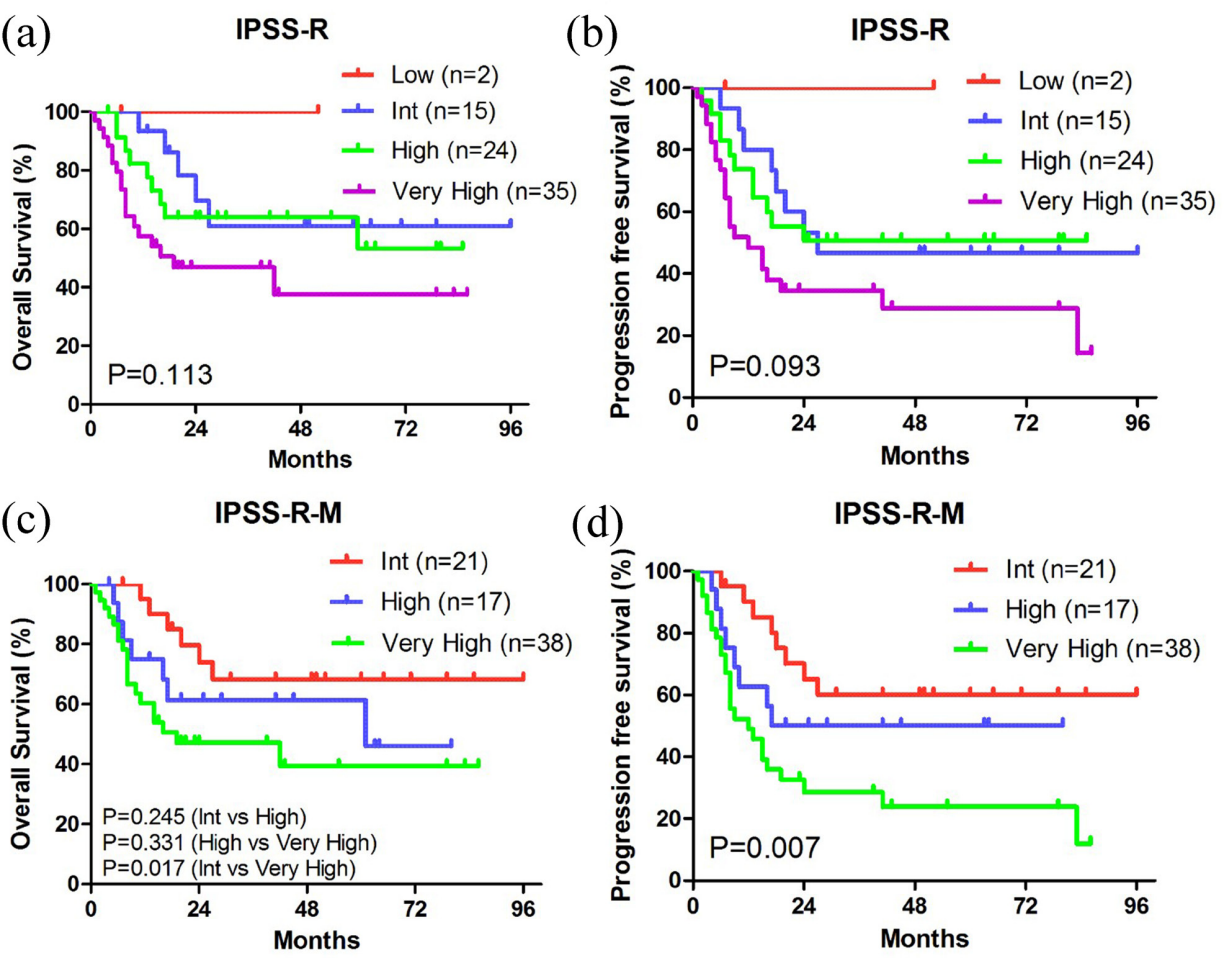
Kaplan-Meier curves of survival according to the IPSS-R and IPSS-R-M systems **(a, c)** Kaplan-Meier curves of OS. **(b, d)** Kaplan-Meier curves of PFS.

The 76 patients with IPSS-R information could be classified into 4 subgroups (low risk, intermediate risk, high risk, and very high risk), with no significant difference in survival, especially between the intermediate and high-risk subgroups (Figure 5a, 5b). The fraction of patients with MDS-related genetic lesions increased to 92.0% when sequencing data of gene mutations (84.0%) were combined with cytogenetics (42.4%), suggesting that molecular markers could be more common. Therefore, we used the integration of molecular marker-based system and IPSS-R to form the new IPSS-R-M system recently proposed by Chen’s group to optimize the prognostic stratification [27]. Based on the IPSS-R-M model, our patients were more accurately classified into 3 prognostic subgroups and the new prognosis data were consistent with previous reports (Figure 5c, 5d) [21, 22].

### A candidate target gene panel for MDS patients

Based on the analysis of the targeted sequencing data of MDS, 17 candidate genes (*DNMT3A*, *GATA1, GATA2*, *TP53*, *RUNX1*, *KRAS, NRAS*, *SRSF2*, *TET2*, *IDH1, IDH2*, *ETV6*, *EZH2*, *BCOR*, *PTPN11*, *STAG2*, *U2AF1*) were identified. These genes have the following characteristics: high frequency, disease specificity, prognostic value and are associated with progressive disease. Combined with a comprehensive literature research for recurrent gene abnormalities in MDS [20-22, 27], especially for genes associated with prognosis, we generated a final candidate panel comprsing 11 genes (*EZH2*, *TET2*, *ASXL1*, *TP53*, *DNMT3A*, *RUNX1*, *ETV6*, *SRSF2*, *U2AF1*, *IDH1, IDH2*) for targeted sequencing in MDS. This 11-gene IPSS-R-M model was preliminarily evaluated on the currently available 76 patients (Supplementary Table 4-5), and our patients were also accurately classified into 3 prognostic subgroups as the same as those of the Chen’s IPSS-M gene panel [27] (Supplementary Figure 3).

## DISCUSSION

In this study, high-throughput deep sequencing of 111 target genes in 125 MDS patients in combination with clinical phenotype analyses identified a comprehensive landscape of genetic lesions in MDS in the Chinese population. Differential mutations were identified in different karyotypes of the MDS patients. Specific gene lesions were also identified in the MDS subgroups defined by the IPSS-R and WHO classifications, highlighting the diagnostic value of these genetic changes. Different pathway mutations could be associated with progression of MDS. Combining with our previous targeted sequencing data in AML, we found that similar genomic signatures might exist between RAEB/MDS-AML and AML, supporting the hypothesis that the two diseases might share common etiologic factors and be affected by similar DNA impairments. The multivariate COX regression analyses revealed that mutation burdens are related to poor survival, supported by the fact that patients with ≥ 2 mutation had shorter PFS. Mutations in *GATA1/GATA2*, *DNMT3A* and *KRAS/NRAS* were also associated with shorter OS. Based on the IPSS-R-M system, these MDS patients could be further stratified. Finally, with a comprehensive analysis and preliminary validation, a new panel of 11 genes was recommended for targeted sequencing in MDS.

Compared with previous studies, several parallels and differences in genomic variants, population coverage and lesions could be observed. The number of identified SNVs and indels were similar between our data (2192 from 61 genes), Haferlach’s and Papaemmanuil’s large scale studies (2764 from 96 genes; 2260 from 43 genes) [21, 22]. Furthermore, this study revealed similar mutational population coverage (84.0%, 105/125) to Haferlach’s study (89.5%, 845/944, p = 0.066) [22], but notably exceeded that in Papaemmanuilʼs data (74.4%, 549/738, p = 0.020) [21].

The spectrum of frequent mutations in this study is similar to that reported in large MDS populations [8, 21, 22]. *ASXL1*, *RUNX1* and *TET2* with mutation frequency >10% in this study were also mutated >10% in studies by Haferlach *et al.* [22] and Bejar *et al.* [8], while Papaemmanuil *et al.* only for *ASXL1* and *TET2* mutation [21]. However, mutation frequency differences could also be observed, possibly because there were more patients with progressive MDS (RAEB, 58%; MDS-AML, 19%) in this study than in the other studies (*i.e.* Papaemmanuil’s: RAEB, 23%; MDS-AML, 5% [21]), and many mutations have various tendency to different MDS subtypes. For example, due to *RUNX1* mutations being associated with an increased risk of progression to AML [8], a higher frequency of this mutation could be observed in our study than in Papaemmanuil’s study (14.4% *vs.* ∼7%) [21]. Moreover, RNA splicing factor mutations (*SRSF2* 5.6%, *U2AF1* 8.0%, *SF3B1* 3.2%, *ZRSR2* 4.0%) were less in our data compared to Haferlach’s study (*SRSF2* 17.6%, *U2AF1* 7.7%, *SF3B1* 32.9%, *ZRSR2* 7.9%) [22], possibly because of differences in the study cohorts.

The relationships between different mutations and karyotypes or IPSS-R or WHO subtypes were interesting when comparing the MDS patients with healthy donators, which has not previously been studied. For example, although mutations in the DNA methylation gene *DNMT3A* were associated with normal karyotype, they were strongly correlated with very high risk subgroup and poor OS [28]. *NRAS* mutations were also prone to normal cytogenetics and were significantly more frequent in MDS-AML, implying they might increase the risk of leukemic transformation in cytogenetically normal MDS [20] and have diagnostic value for MDS-AML. *ETV6* mutations cause loss of function, thereby resulting in incapability of transcription repression [20] and exhibited dominant negative effects on survival [23], regardless of the normalcy of karyotypes in our study. Results also indicated that *RUNX1* mutations are more common in high-risk MDS patients with short survival, which was consistent with previous findings [29]. In contrast, although in our cohort *EZH2* mutations were more common in high-risk patients and had no prognostic value, a previous study identified a group of lower-risk MDS patients with *EZH2* mutations and worse-than-expected prognosis [24]. Mutations in the splicing factor gene *SRSF2* were notably rich in RAEB in our study and might negatively affect prognosis based on previous studies [30], different from a report of favorable prognosis by Bejar *et al.* [24]. The discrepancies above might result from diversities of the study populations, sequencing methods and scopes of target gene among these studies. Nevertheless, these differences should inspire more exploration using systems suitable for varying cohorts with consistent methodology and clinically significant genes. We proposed a panel of 11 genes for targeted sequencing as a step to address this problem. Future comprehensive studies are warranted to validate this panel.

Patterns of genomic lesions are considered as genomic signatures in cancer [31, 32]. In this study, we found that nucleotide substitutions were the main type of mutations with transitions being the dominant form. The most prevalent changes were C→T transitions, followed by G→A/A→G/T→C transitions, which was similar to the pattern seen in MDS and AML in previous exome sequencing/whole-genome sequencing studies [14, 27, 33, 34]. These results confirmed that the pattern of genomic lesions was a signature of MDS, regardless of the sequencing method, and that there is a close relationship between MDS and AML in terms of pathogenesis [19].

To explore the molecular pathogenesis of MDS progression [20, 35], the target genes were grouped into 7 functional pathways, as previously reported [22]. The three most frequently mutated pathways were transcription (58.4%), epigenetic modification (53.6%) and signal transduction (52.0%). However, a higher mutation frequency in RNA splicing (64%) and lower frequencies in transcription (28%) and signal transduction (15%) were observed in Haferlach’s data [22]. Additionally, mutated genes in the epigenetic modification and signal transduction pathways appeared in each subtype of MDS, consistent with common molecular mechanisms of MDS reported in several studies [20]. DNA methylation-related mutations occurred early in disease evolution and could trigger MDS through cooperation with other mutations frequently found in hematologic malignancies [10, 20]. Mutations in genes involved in signal transduction were often associated with the pathogenesis of MDS and contributed to the progression of MDS [20, 36]. Furthermore, signal transduction was the most commonly mutated pathway in RCMD but rarely mutated in RAEB (p = 0.02), whereas mutations in transcription reached a maximum ratio in RAEB and also accounted for a large proportion in MDS-AML. Mutations affecting the cohesion pathway were only found in RAEB and MDS-AML. All these changes might underlie the evolution from non-progressive MDS to progressive MDS. Finally, RAEB and MDS-AML had fairly similar mutational profiles, implying a mechanism correlation between the two diseases [37].

The molecular mechanisms of progressive MDS and *de novo* AML require joint actions of factors involved in epigenetic modification, signal transduction, RNA splicing and cohesion pathways [10, 27], the absence of mutations in DNA repair and cell cycle regulation as well as the increase of transcription-related mutations (other than those in *NPM1* and *WT1*), which might indicate a progressive expansion capacity of abnormal clones in *de novo* AML [27]. In particular, this notion is consistent with previous results which have shown that *NPM1* is rarely mutated in MDS [27, 35]. Hence, the molecular discrepancies used for distinguishing progressive MDS and *de novo* AML might be found in the functional pathways affected by genetic lesions, which could possibly aid in identifying patients with progressive disease before symptoms associated with higher-risk disease are manifested.

DNA methylation might play an important role in the pathogenesis of MDS. In parallel, MDS patients appear to benefit from treatments with HMAs [38–40]. However, it remains unclear how mutations in DNA methylation can be utilized to predict benefits of HMAs [41]. We found that patients with mutations in DNA methylation carried more unfavorable features. However, the OS and PFS of these patients were not significantly different from those without mutations in DNA methylation. We noted that more patients in the former group were treated with HMAs, which could have improved the situations in this group, and might, therefore, explain the similar OS and PFS aforementioned [39, 42, 43]. Nevertheless, further study is warranted to clarify the use of mutational information in DNA methylation as a reference for HMAs treatment.

Previous analyses of survival suggested that heavier mutational burdens might be associated with poorer prognosis [21]. We found that RAEB patients had a considerably higher average number of mutations compared to RCMD patients. Accordingly RAEB patients had reduced PFS (p < 0.05). In addition, the average number of mutations in MDS-AML was the highest in our study and these patients had the lowest survival (p < 0.05). We also found that patients with ≥ 2 co-occurring mutations had significantly shorter PFS than those with <2 mutations (HR, 3.364; 95%CI, 1.428-7.925). The correlation between mutational burdens in MDS subtypes and survival was also consistent with Chen’s findings [27].

In our COX regression analysis, mutations in *GATA1/GATA2*, *DNMT3A* and *KRAS/NRAS* were related to shorter OS (p < 0.05), consistent with previous studies [9, 20], Interestingly, patients harboring *IDH1/IDH2* mutations had prolonged PFS (p < 0.05). Previous studies had controversial findings about how *IDH1/IDH2* mutations affected the prognosis of MDS patients [23, 44]. A recent meta-analysis including 1782 patients showed poor survival in patients with mutant *IDH1/IDH2* [45]. It is worth noting that our results were limited by the small sample size, probably explaining the discrepancies between our findings and certain previous studies [21, 22]. Based on the novel IPSS-R-M system derived from Chen’s study [27], our patients were further stratified into higher and lower risk groups, which could potentially be used to predict prognosis of MDS patients more accurately, identify disease progression and guide refined therapeutic strategies. Compared to Chen’s gene panel (21-gene panel) [27], although the 11-gene panel only included half the number of the genes, it exhibited equal prognostic value with the same reasonable classification (Figure 5, Supplementary Figure 3). This panel remains to be validated in large-scale patient studies and could be useful in clinical application.

In conclusion, we performed NGS to screen 111 genes in 125 patients with MDS, analyzed the mutational profiles and prognosis of these patients and compared our findings with previous studies. Our study provides new insights into the underlying genetic mechanisms of Chinese MDS patients. We proposed a panel of 11 genes that might be used for the genetic profiling of MDS and guide the development of individualized therapies. This panel remains to be validated in future larger-scale studies.

## MATERIALS AND METHODS

### Ethics statement

The study protocol was approved by the Ethics Committee of the General Hospital of Chinese People’s Liberation Army, and was conducted in accordance with the Declaration of Helsinki. Written informed consents were obtained from all participants and/or their legal guardian.

### Patients

A total of 125 newly diagnosed MDS patients and 81 healthy volunteers who visited our Hematology Department and Bone Transplantation Center between August 2008 and September 2014 were enrolled in the study (Table 1). 5 ml EDTA-anticoagulated bone marrow samples from MDS patients or peripheral blood samples from healthy volunteers were collected. Genomic DNAs were extractedusing a DNA Purification Kit (Promega, USA). The charts, electronic medical records and laboratory records were reviewed by two trained hematologists. Data on demographic characteristics, diagnosis and treatment of MDS, survival status, etc. were collected and recorded in the case report form. Finally, all the data were put into an Excel database and further evaluated by a third trained hematologist.

The diagnosis and classification of MDS were based on the WHO 2008 classification, and MDS-AML was defined as AML with multilineage dysplasia following MDS [46]. Of the 125 patients, 42 were women and 83 were men. The median age was 49 years (range, 14-82 years). Refractory anemia with excess blasts (RAEB) was the main subtype (58%, 72/125) and 24 cases (19%) progressed to MDS-AML. From the 108 patients with available outcome data, 50 (46%) had abnormal cytogenetics and 74 (69%) cases were in the intermediate or above risk group. The median follow-up was 18 months for OS and 13 months for PFS (Table 1).

### Targeted-sequencing and study controls

The sequencing panel targets ∼250 kb genomic content that covers the entire coding sequences of 111 genes relevant to the pathogenesis of MDS. In this panel, 42 genes originated from the hematopoietic diagnosis and treatment guidelines published by the National Comprehensive Cancer Network (NCCN) and WHO, and 69 genes were selected based on a literature search for recurrent gene abnormalities in MDS (Supplementary Table 6).

Genomic DNA extracted from bone marrow (MDS patients, n = 125) or peripheral blood (healthy volunteers, n = 81) was examined for mutations in 111 known genes by targeted sequencing. Germline DNAs were not available. In our previous report [26], the reliability of healthy adults as controls has been proved compared to the patients’ matched saliva controls. Therefore, 81 healthy volunteers (females and males: 42 and 39, respectively; median age: 29 years, range 23-56 years) were used as controls to remove germline and harmless mutations. If a mutation occurred in at least 1 of the 81 healthy individuals, it would be removed (details in Supplementary Method).

NimbelGenSeqCap EZ Choice was used according to the manufacturer’s protocol with modifications. Multiplexed libraries were sequenced using 100-bp paired-end runs on an Illumina HiSeq 2500. Reads were aligned using the Burrows-Wheeler alignment (BWA) tool to human genomic reference sequences (HG19, NCBI built 37) [47]. To identify single nucleotide polymorphisms (SNPs) and short insertions and deletions (INDELs), MuTect2 with recommended parameters was performed [48]. All mutations were annotated by the ANNOVAR software using some resources (details in Supplementary Method) [49]. A subset of somatic mutations was randomly selected for validation using Sanger sequencing (Supplementary Table 7). Cell line dilution was prepared for evaluation of sensitivity and specificity (details in Supplementary Method, Supplementary Table 8, Supplementary Figure 4).

### Statistical analyses

The demographics and characteristics were summarized using descriptive statistics. Student’s *t* test or Mann-Whitney U-test were used to compare the differences in continuous variables. Analysis of frequencies was performed using the Fisher’s exact test or Pearson’s χ2 test. Survival analysis was performed by Kaplan-Meier method and a Cox proportional hazard model was used to assess the prognostic significance of the clinical variables. OS was defined as the time from diagnosis to death from any cause since last follow-up. PFS was defined as the time from diagnosis to progression (the classification of MDS at diagnosis progressing to the next stage, *i.e.* RCMD to RAEB1, or AML or death from any cause), or last follow-up. A two-sided p-value < 0.05 was considered statistically significant. All statistical analyses were performed with the SPSS software version 19.0 (IBM Corp., Armonk, NY, USA).

## SUPPLEMENTARY MATERIALS FIGURES AND TABLES








