# Supplementary material for: Implications of mutational spectrum in myelodysplastic syndromes based on targeted next-generation sequencing
Source: Oncotarget. 2017 Jul 27;8(47):82475–90. doi: 10.18632/oncotarget.19628 (PMC5669905; doi:10.18632/oncotarget.19628)
Supplement: Supplementary file 1 [file oncotarget-08-82475-s001.pdf]

Implications of mutational spectrum in myelodysplastic syndromes based on targeted next-generation sequencing

SUPPLEMENTARY MATERIALS

Illumina library construction and sequencing

Indexed Illumina NGS libraries were prepared from 125 MDS bone marrow samples and 81 peripheral blood leukocyte genomic DNA samples. 1 μg DNA from each sample was sheared for library construction with a Bioruptor instrument using the recommended settings for 200-bp fragments. NGS libraries were constructed following the Illumina standard DNA sample preparation protocol using DNA Polymerase. After hybrid selection, captured DNA fragments were amplified with 13 cycles of PCR using 1× KAPA HiFi Hot Start Ready Mix and 0.4 μM Illumina backbone oligonucleotides in 50-μL reactions. The reactions were pooled and quantified with QPCR. Multiplexed libraries were sequenced using 100-bp paired-end runs on an Illumina Hiseq 2500.

Mutation detection pipeline

For 125 MDS, the SCARF file was converted to FASTQ format by Casava software version 1.8 (Illumina). Raw sequence reads were filtered with an indigenous program. We treated bases with Phred Quality Score (Q) ≤ 5 as low quality bases. Reads with more than 5% N bases which can not be determined to be A, T, G or C, or in which more than 50% bases had Q ≤ 5, or containing adapters were eliminated. After filter the median percentage of bases with Q over 30 in all was 93.059 (max: 96.241; min: 90.808). The remaining reads were aligned using a Burrows-Wheeler alignment (BWA-0.7.5a) tool to the human genomic reference sequences (HG19, NCBI built 37) with certain parameters (mem –t 10 –k 32 -M) [1]. To decrease PCR duplication bias, the resulting Bam files were processed with Samtools. Only unique reads were delivered for analyses. For identification of SNP and indel, MuTect2 with recommended parameters was performed [2]. All mutations were annotated by ANNOVAR software [3] using the following resources: all annotated transcripts in RefSeq Gene; known constitutional polymorphisms as reported in known human variation databases, such as 1000 Genomes (20130308) and the Exome Aggregation Consortium (ExAC: 20151129) were download from ANNOVAR and dbSNP was v135 [4]; known somatic variations in myeloid and other malignancies as reported in COSMIC v70 [5]. To identify high-confidence somatic variants in all MDS samples in the absence of matched control samples, the following criteria were used: removal of all variants within intronic, UTR and intergenic regions, and retention of only nonsynonymous, frameshift and stop-gain mutations in exonic regions; removal of all variants present in at least 1 of 81 healthy individuals; removal of all variants with one of the following features in MuTect results: read depth less than 4, Phred-scaled *p*-value using Fisher's exact test to detect strand bias more than 60, variant confidence/quality by depth below 2, mapping quality lower than 40, Alt/Ref read mapping qualities no more than -12.5 and Alt/Ref read position bias not over -8.

Removal of harmless mutations

Because we lacked matched normal samples, somatic mutations could not be selected by comparing tumor and matched normal sample. Thus, a series of steps were used to remove germline mutations and harmless mutations. Mutations were removed unless they satisfied all of the following conditions: the mutation depth was larger than 4; occurred in an exonic region; the mutation function was not "synonymous SNV"; the annotation from ClinVar was not "benign" or the mutation did not appear in a dbSNP135 or the 1000 Genomes Project (2012 Feb) database.

Evaluation of sensitivity and specificity of cell line dilution data

To evaluate the MuTect pipeline in mixed sample, cell dilution data were used. Assuming that 0 and 100% solutions of Kasumi-1 cells were accurate, we hypothesized that at each dilution of the cells we could calculate true SNPs and false-positive SNPs. The reads number that supports the mutation was considered to be the only requirement for mutation. Sequencing results from four different depths indicated that over 90% of the sensitivity could be gained in more than 3% of the mutations at 800× sequencing depths. In contrast, false-positives were detected at 0% and 100% K562 cell solutions. With 800× sequencing depth, false-positives were less than 2%. Thus, mutation detection is efficient with over 800× sequencing coverage.

REFERENCES

1. Li H, Durbin R. Fast and accurate short read alignment with Burrows-Wheeler transform. Bioinformatics. 2009; 25:1754-60.
2. Cibulskis K, Lawrence MS, Carter SL, Sivachenko A, Jaffe D, Sougnez C, Gabriel S, Meyerson M, Lander ES, Getz G. Sensitive detection of somatic point mutations in impure and heterogeneous cancer samples. Nat Biotechnol. 2013; 31:213-9.
3. Wang K, Li M, Hakonarson H. ANNOVAR: functional annotation of genetic variants from high-throughput sequencing data. Nucleic Acids Res. 2010; 38:e164.
4. Joober R. The 1000 Genomes Project: deep genomic sequencing waiting for deep psychiatric phenotyping. J Psychiatry Neurosci. 2011; 36:147-9.
5. Forbes SA, Bindal N, Bamford S, Cole C, Kok CY, Beare D, Jia M, Shepherd R, Leung K, Menzies A, Teague JW, Campbell PJ, Stratton MR, Futreal PA. COSMIC: mining complete cancer genomes in the Catalogue of Somatic Mutations in Cancer. Nucleic Acids Res. 2011; 39:D945-50.

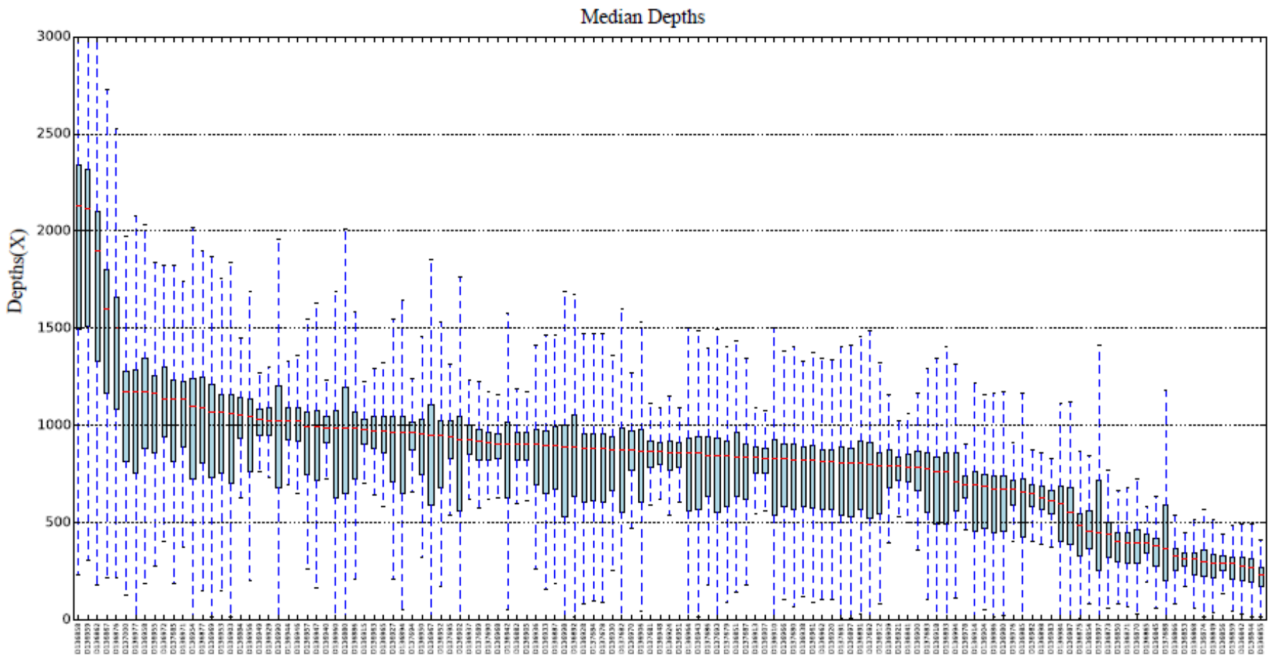

Supplementary Figure 1: Quality control chart of the 125 patients' NGS data. X axis: Sequencing sample. Y axis: Sequencing depth.

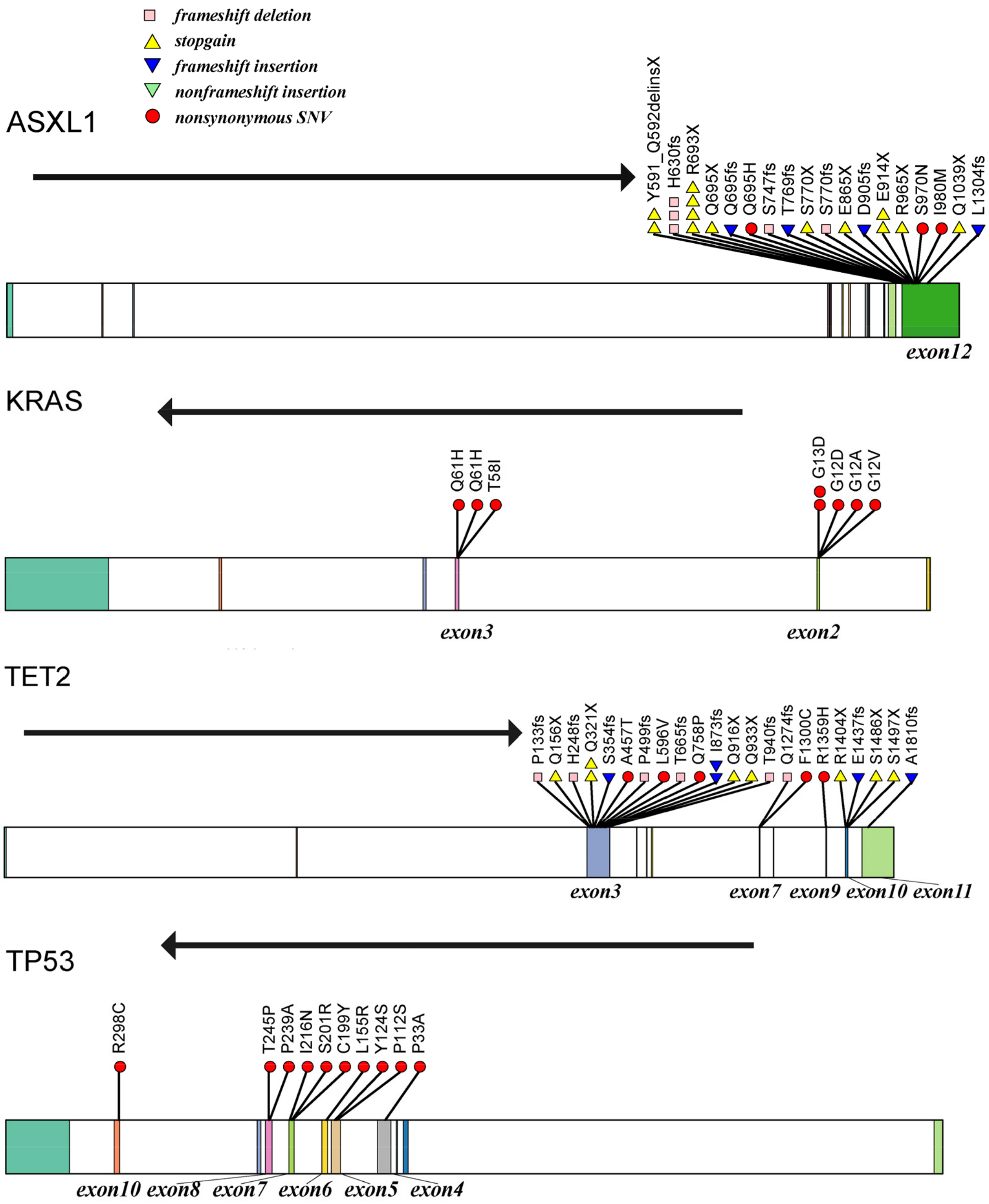

Supplementary Figure 2: Positions and types of mutations identified in this study.

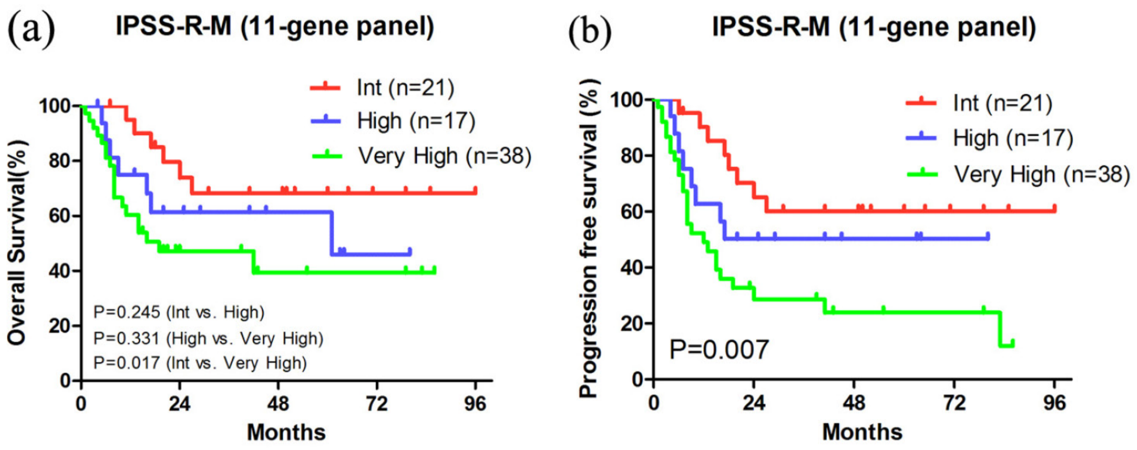

Supplementary Figure 3: Kaplan-Meier curves of survival according to the IPSS-R-M system (11-gene panel). (a) Kaplan-Meier curves of OS. **(b)** Kaplan-Meier curves of PFS.

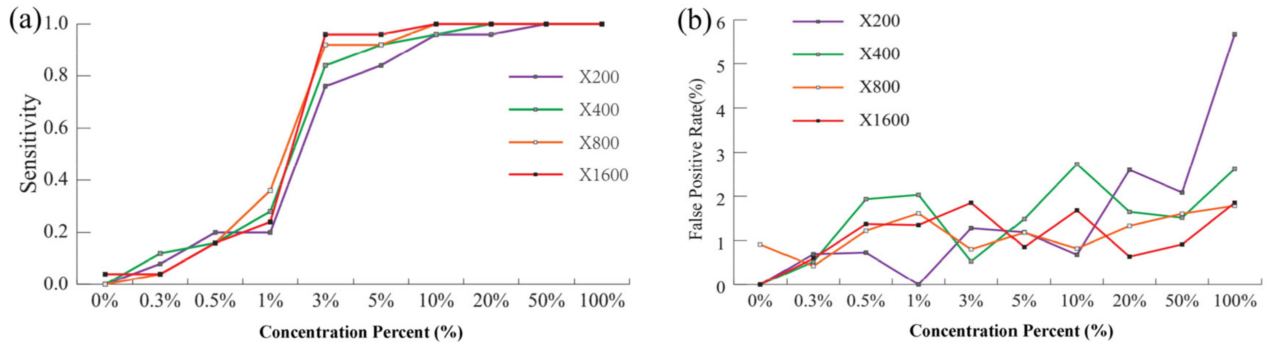

Supplementary Figure 4: Sensitivity and false-positive mutation detection rates. **(a)** In Kasumi-1 and K562 cell lines, mutation sensitivity can be calculated and more than 90% of the unique mutations within Kasumi-1 can be detected. **(b)** The false-positive rate revealed that more than 98% mutations were consistent with the Kasumi-1 and K562 cell line data.

Supplementary Table 1: The detailed NGS data generation of the 125 patients

See Supplementary File 1

Supplementary Table 2: The list of 61 genes identified in this study

See Supplementary File 2

Supplementary Table 3: The list of gene mutations detected in the study

See Supplementary File 3

Supplementary Table 4: IPSS-R-M prognostic scoring system (11-gene panel)

Prognostic variable	Score						
	**0**	**0.5**	**1**	**1.5**	**2**	**3**	**4**
Cytogenetics[#]	Very Good	-	Good	-	Intermediate	Poor	Very Poor
Bone marrow blast (%)	≤ 2		>2-<5	-	5-10	>10	-
Hemoglobin (g/L)	≥100	-	80-<100	<80	-	-	-
Platelets (×10^9/L)	≥100	50-<100	<50	-	-	-	-
ANC (×10^9/L)	≥0.8	<0.8	-	-	-	-	-
Number of mutations[*]	0	1	≥2	High risk mutation[**]			

[#]Very Good: -Y, del(11q); Good: normal, del(5q), del(12p), del(20q), double including del(5q); Intermediate: del(7q), +8, +19, i(17q), any other single or double independent clones; Poor: -7, inv(3)/t(3q)/del(3q), double including -7/del(7q), complex(3 abnormalities); Very Poor: >3 abnormalities.

[*]Mutations in 11 genes: *EZH2, TET2, ASXL1, TP53, DNMT3A, RUNX1, ETV6, SRSF2, U2AF1, IDH1, IDH2*

[**]Any mutation of *EZH2, RUNX1, IDH1, IDH2* and *TP53*.

Supplementary Table 5: IPSS-R-M prognostic risk categories/scores (11-gene panel)

Risk category	Patients, *n*	Risk scores	3-year OS (%)	3-year PFS (%)
Intermediate	21	>3.5-5.0	68.3±10.8	60.2±10.9
High	17	>5.0-6.5	61.4±12.4	50.2±12.5
Very High	38	>6.5	47.2±8.7	28.6±8.2

[#]IPSS-R-M risk score = IPSS-R risk score + 0.5.

Supplementary Table 6: The list of 111 genes in the panel

ABCB1	BRAF	CSF3R	FBXW7	JAK1	MPL	PIGA	STAT5A
ABCC3	CACNA1E	CTLA4	FLT3	JAK2	MTHFR	PIK3CA	STAT5B
ABL1	CALR	CUX1	CSF1R	JAK3	MYD88	PTEN	SYK
AKT2	CARD11	CYP2C19	GATA1	KDM6A	NF2	PTPN11	TCF3
AKT3	CBL	CYP3A4	GATA2	KIT	NOTCH1	RAD21	TERC
AMER1	CBLB	DIS3	GNAS	KMT2A	NOTCH2	RB1	TET2
RUNX1	CBLC	DNAH9	GSTM1	KMT2C	NPM1	SETBP1	TP53
APC	CCND1	DNMT3A	GSTP1	SETD2	NQO1	SF3B1	TPMT
ASXL1	CD79B	EGFR	HRAS	KRAS	NRAS	SH2B3	TRAF3
ATM	CDA	ERCC1	ID3	MAP2K4	NT5C2	SMAD4	U2AF1
ATRX	CDKN2A	ERG	IDH1	MAP3K7	NTRK1	SMC1A	WT1
BCL2	CEBPA	ETV6	IDH2	MDM2	NTRK2	SMC3	XRCC1
BCOR	CREBBP	EZH2	IKZF1	MEF2B	PDGFRA	SRSF2	ZRSR2
BCORL1	CRLF2	FAM46C	IL7R	MLH1	PHF6	STAG2	

Supplementary Table 7: The validation of gene mutations by Sanger sequencing

See Supplementary File 3

Supplementary Table 8: Cell line dilution data

ID	Component	
	Kasumi-1 (*AML1-ETO*+)	K562 (*BCR-ABL*+)
HC14AN00001	0%	100%
HC14AN00002	3‰	99.7%
HC14AN00003	5‰	99.5%
HC14AN00004	1%	99%
HC14AN00005	3%	97%
HC14AN00006	5%	95%
HC14AN00007	10%	90%
HC14AN00008	20%	80%
HC14AN00009	50%	50%
HC14AN00010	100%	0%

To evaluate the performance of a mutation detection method, Kasumi-1 and K562 cells were diluted at different ratios.
