# Supplementary material for: Implications of mutational spectrum in myelodysplastic syndromes based on targeted next-generation sequencing
Source: Oncotarget. 2017 Jul 27;8(47):82475–90. doi: 10.18632/oncotarget.19628 (PMC5669905; doi:10.18632/oncotarget.19628)
Supplement: Supplementary file 4 [file oncotarget-08-82475-s004.doc]

**Supplementary Table 3: The list of gene mutations detected in the study**.

| **Function** | **Gene** | **MDS**  **(n=125)** | **Freq**  **(MDS)** | **RCMD**  **(n=21)** | **Freq**  **(RCMD)** | **RAEB**  **(n=73)** | **Freq**  **(RAEB)** | **MDS-AML**  **( n=23)** | **Freq**  **(MDS-AML)** |
| --- | --- | --- | --- | --- | --- | --- | --- | --- | --- |
| Cohesion | STAG2 | 10 | 11.2% | 0 | 0 | 6 | 13.7% | 4 | 17.4%  (4/23) |
|  | RAD21-AS1 | 1 |  | 0 |  | 1 |  | 0 |  |
|  | RAD21 | 3 |  | 0 |  | 3 |  | 0 |  |
| Epigenetic  modification | TET2 | 15 | 53.6% | 0 | 33.3% | 10 | 54.8% | 5 | 73.9%  (17/23) |
|  | SETD2 | 3 |  | 1 |  | 2 |  | 0 |  |
|  | DNMT3A | 10 |  | 1 |  | 5 |  | 4 |  |
|  | IDH1 | 4 |  | 0 |  | 4 |  | 0 |  |
|  | IDH2 | 4 |  | 1 |  | 3 |  | 0 |  |
|  | KMT2A | 1 |  | 0 |  | 1 |  | 0 |  |
|  | ASXL1 | 21 |  | 2 |  | 11 |  | 8 |  |
|  | EZH2 | 7 |  | 2 |  | 4 |  | 0 |  |
|  | KDM6A | 2 |  | 0 |  | 0 |  | 0 |  |
| RNA splicing | SRSF2 | 7 | 20.8% | 0 | 9.5% | 6 | 26% | 1 | 17.4%  (4/23) |
|  | U2AF1 | 10 |  | 2 |  | 7 |  | 1 |  |
|  | SF3B1 | 4 |  | 0 |  | 2 |  | 1 |  |
|  | ZRSR2 | 5 |  | 0 |  | 4 |  | 1 |  |
| Transcription | CEBPA | 7 | 58.4% | 1 | 14.3% | 4 | 69.9% | 2 | 73.9%  (17/23) |
|  | RUNX1 | 18 |  | 0 |  | 12 |  | 6 |  |
|  | BCOR | 9 |  | 0 |  | 7 |  | 2 |  |
|  | BCORL1 | 4 |  | 0 |  | 2 |  | 1 |  |
|  | PHF6 | 3 |  | 0 |  | 3 |  | 0 |  |
|  | GATA1 | 1 |  | 0 |  | 1 |  | 0 |  |
|  | GATA2 | 6 |  | 0 |  | 3 |  | 3 |  |
|  | ETV6 | 9 |  | 1 |  | 6 |  | 2 |  |
|  | IKZF1 | 1 |  | 0 |  | 1 |  | 0 |  |
|  | NPM1 | 2 |  | 0 |  | 1 |  | 1 |  |
|  | TP53 | 7 |  | 1 |  | 5 |  | 0 |  |
|  | WT1 | 1 |  | 0 |  | 1 |  | 0 |  |
|  | ABCC3 | 1 |  | 0 |  | 1 |  | 0 |  |
|  | ID3 | 1 |  | 0 |  | 1 |  | 0 |  |
|  | STAT5A | 1 |  | 0 |  | 1 |  | 0 |  |
|  | ERG | 1 |  | 0 |  | 1 |  | 0 |  |
|  | CYP3A4 | 1 |  | 0 |  | 1 |  | 0 |  |
| Signal transduction | JAK2 | 6 | 52% | 2 | 47.6% | 4 | 47.9% | 0 | 73.9%  (17/23) |
|  | NOTCH1 | 1 |  | 0 |  | 1 |  | 0 |  |
|  | NOTCH2 | 6 |  | 3 |  | 3 |  | 0 |  |
|  | PIGA | 3 |  | 2 |  | 0 |  | 0 |  |
|  | KRAS | 6 |  | 0 |  | 4 |  | 2 |  |
|  | NRAS | 5 |  | 0 |  | 1 |  | 4 |  |
|  | CBL | 2 |  | 0 |  | 2 |  | 0 |  |
|  | AMER1 | 2 |  | 1 |  | 1 |  | 0 |  |
|  | CUX1 | 5 |  | 1 |  | 4 |  | 0 |  |
|  | CREBBP | 1 |  | 0 |  | 1 |  | 0 |  |
|  | GNAS | 3 |  | 0 |  | 3 |  | 0 |  |
|  | KIT | 1 |  | 0 |  | 0 |  | 1 |  |
|  | CARD11 | 1 |  | 0 |  | 1 |  | 0 |  |
|  | FLT3-ITD | 5 |  | 0 |  | 2 |  | 3 |  |
|  | MAP3K7 | 2 |  | 0 |  | 1 |  | 1 |  |
|  | PTPN11 | 6 |  | 0 |  | 2 |  | 4 |  |
|  | CSF3R | 4 |  | 0 |  | 3 |  | 1 |  |
|  | CBLC | 1 |  | 0 |  | 0 |  | 0 |  |
|  | AKT2 | 1 |  | 1 |  | 0 |  | 0 |  |
|  | EGFR-AS1 | 1 |  | 0 |  | 0 |  | 0 |  |
|  | SH2B3 | 3 |  | 0 |  | 2 |  | 1 |  |
| DNA repair | SETBP1 | 9 | 10.4% | 1 | 4.8% | 6 | 13.7% | 1 | 4.3%  (1/23) |
|  | ATM | 4 |  | 0 |  | 4 |  | 0 |  |
| Cell cycle regulation | CDKN2A | 1 | 7.2% | 0 | 4.8% | 1 | 9.6% | 0 | 4.3%  (1/23) |
|  | DIS3 | 3 |  | 1 |  | 2 |  | 0 |  |
|  | APC | 2 |  | 0 |  | 2 |  | 0 |  |
|  | FBXW7 | 1 |  | 0 |  | 1 |  | 0 |  |
|  | RB1 | 2 |  | 0 |  | 1 |  | 1 |  |

**Supplementary Table 7: The validation of gene mutations by Sanger sequencing**.

| Gene  name | Sample  ID | Version | Position | Geno-  type | Backward  15 bases | Sanger  results | Valid-ation |
| --- | --- | --- | --- | --- | --- | --- | --- |
| DNMT3A | D136913 | Hg19 | 25457242 | G/A | GGCTCATGTTGGAGA | G/A | Yes |
| DNMT3A | D136940 | Hg19 | 25457242 | G/A | GGCTCATGTTGGAGA | G/A | Yes |
| DNMT3A | D136956 | Hg19 | 25457242 | G/A | GGCTCATGTTGGAGA | G/A | Yes |
| DNMT3A | D136954 | Hg19 | 25457243 | C/T | GCTCATGTTGGAGAC | C/T | Yes |
| DNMT3A | D136924 | Hg19 | 25463271 | C/G | CATCATGCAGGAGGC | C/G | Yes |
| DNMT3A | D136951 | Hg19 | 25463308 | C/T | GCCAGTGCCCTCTGA | C/T | Yes |
| DNMT3A | D137692 | Hg19 | 25467158 | C/T | GGCAGCCCCCGGCCC | C/T | Yes |
| DNMT3A | D136953 | Hg19 | 25467493 | A/G | ACTGGTACGCACACT | A/G | Yes |
| DNMT3A | D136977 | Hg19 | 25467493 | A/G | ACTGGTACGCACACT | A/G | Yes |
| KRAS | D136870 | Hg19 | 25380275 | A/T | TGACCTGCTGTGTCG | A/T | Yes |
| KRAS | D136967 | Hg19 | 25398281 | G/A | CACCAGCTCCAACTA | G/A | Yes |
| KRAS | D136998 | Hg19 | 25398281 | G/A | CACCAGCTCCAACTA | G/A | Yes |
| KRAS | D136972 | Hg19 | 25398284 | G/A | CAGCTCCAACTACCA | G/A | Yes |
| KRAS | D137693 | Hg19 | 25398284 | G/T | CACCAGCTCCAACTA | G/T | Yes |
| NRAS | D136903 | Hg19 | 115256529 | A/T | GTCCAGCTGTATCCA | A/T | Yes |
| NRAS | D137690 | Hg19 | 115258744 | G/A | CACCTGCTCCAACCA | G/A | Yes |
| SETBP1 | D136977 | Hg19 | 42529970 | G/C | GTCCACCAACTCTGA | G/C | Yes |
| SETBP1 | D136844 | Hg19 | 42530177 | C/T | ATCCCCAAGCAGCCA | C/T | Yes |
| SETBP1 | D136970 | Hg19 | 42531634 | C/A | ACCCACTTTCAACAC | A/T | Yes |
| SETBP1 | D136870 | Hg19 | 42531907 | G/A | ACAGCGGCATTGGGA | G/A | Yes |
| SETBP1 | D136967 | Hg19 | 42531907 | G/A | ACAGCGGCATTGGGA | G/A | Yes |
| SETBP1 | D136998 | Hg19 | 42531907 | G/A | ACAGCGGCATTGGGA | G/A | Yes |
| SETBP1 | D136898 | Hg19 | 42531913 | G/A | GCATTGGGACAGACA |  | NA* |
| SETBP1 | D136912 | Hg19 | 42531925 | G/A | ACAACAACAGCACTT | G/A | Yes |
| SRSF2 | D136943 | Hg19 | 74732936 | 284_307del | GCGGCTGTGGTGTGA | 284_307del | Yes) |
| SRSF2 | D136850 | Hg19 | 74732959 | C/A | GGCGGCCGTAGCGCG | C/A | Yes |
| SRSF2 | D136874 | Hg19 | 74732959 | C/A | GGCGGCCGTAGCGCG | C/A | Yes |
| SRSF2 | D136980 | Hg19 | 74732959 | C/A | GGCGGCCGTAGCGCG | C/A | Yes |
| SRSF2 | D136997 | Hg19 | 74732959 | C/A | GGCGGCCGTAGCGCG | C/A | Yes |
| SRSF2 | D136952 | Hg19 | 74732959 | C/G | GGCGGCCGTAGCGCG | C/G | Yes |
| SRSF2 | D136849 | Hg19 | 74732959 | C/T | GGCGGCCGTAGCGCG | C/T | Yes |
| STAG2 | D136972 | Hg19 | 123164936 | 249delG | TTGTTTGAAGTTGTT | 249delG | Yes |
| STAG2 | D136859 | Hg19 | 123179030 | 479_483del | ACTTACCATGGCTGG | 479_483del | Yes |
| STAG2 | D136860 | Hg19 | 123179030 | 479_483del | ACTTACCATGGCTGG |  | NA* |
| STAG2 | D137694 | Hg19 | 123181296 | 760delA | AAATGATTGGAAAAC | 760delA | Yes |
| STAG2 | D137687 | Hg19 | 123182911 | 876_877insTT | TTTGTACATAGATAC | 876_877insTT | Yes |
| STAG2 | D136943 | Hg19 | 123191721 | 1311dupC | CAGTCGTAGAGATCC | 1311dupC | Yes |
| TP53 | D136921 | Hg19 | 7574018 | C/T | CTCACGCCCACGGAT | C/T | Yes |
| TP53 | D136990 | Hg19 | 7577088 | A/C | GCGCCGGTCTCTCCC | A/C | Yes |
| TP53 | D136990 | Hg19 | 7577517 | T/A | TGATGGTGAGGATGG | T/A | Yes |
| TP53 | D136971 | Hg19 | 7577561 | T/A | CTGTTACACATGTAG | T/A | Yes |
| TP53 | D136909 | Hg19 | 7577568 | G/A | ACATGTAGTTGTAGT | G/A | Yes |
| TP53 | D136956 | Hg19 | 7578268 | T/G | GATGCTGAGGAGGGG | T/G | Yes |
| TP53 | D136971 | Hg19 | 7578442 | A/C | AGATGGCCATGGCGC | A/C | Yes |
| TP53 | D136914 | Hg19 | 7579473 | C/G | GGGAGCAGCCTCTGG | C/G | Yes |
| U2AF1 | D136875 | Hg19 | 44514769 | 477_478ins  TATGAG | CTCATACTGACGGCA | 477_478ins  TATGAG | Yes |
| U2AF1 | D136898 | Hg19 | 42531913 | A/G | GCATTGGGACAGACA |  | NA* |
| U2AF1 | D136884 | Hg19 | 44524456 | C/A | AGCACCTGTCTCCAT | C/A | Yes |
| U2AF1 | D136944 | Hg19 | 44524456 | C/A | AGCACCTGTCTCCAT | C/A | Yes |
| U2AF1 | D136949 | Hg19 | 44524456 | C/A | AGCACCTGTCTCCAT | C/A | Yes |
| U2AF1 | D136959 | Hg19 | 44524456 | C/A | AGCACCTGTCTCCAT |  | NA* |
| U2AF1 | D137685 | Hg19 | 44524456 | C/A | AGCACCTGTCTCCAT | C/A | Yes |
| U2AF1 | D137689 | Hg19 | 44524456 | C/A | AGCACCTGTCTCCAT | C/A | Yes |

*not available due to lack of samples.
